# The impact of intimate partner violence on adverse birth outcomes in 20 sub-Saharan African countries: propensity score matching analysis

**DOI:** 10.3389/fgwh.2024.1420422

**Published:** 2024-10-28

**Authors:** Angwach Abrham Asnake, Beminate Lemma Seifu, Alemayehu Kasu Gebrehana, Asaye Alamneh Gebeyehu, Amanuel Yosef Gebrekidan, Afework Alemu Lombebo, Amanuel Alemu Abajobir

**Affiliations:** ^1^ Department of Epidemiology and Biostatistics, School of Public Health, College of Health Sciences and Medicine, Wolaita Sodo University, Wolaita Sodo, Ethiopia; ^2^ Department of Public Health, College of Medicine and Health Sciences, Samara University, Samara, Ethiopia; ^3^ Department of Midwifery, College of Medicine and Health Sciences, Salale University, Salale, Ethiopia; ^4^ Department of Public Health, College of Health Science, Debre Tabor University, Debre Tabor, Ethiopia; ^5^ School of Public Health, College of Health Sciences and Medicine, Wolaita Sodo University, Wolaita Sodo, Ethiopia; ^6^ School of Medicine, College of Health Science and Medicine, Wolaita Sodo University, Wolaita Sodo, Ethiopia; ^7^ African Population and Health Research Center, Nairobi, Kenya; ^8^ School of Public Health, The University of Queensland, Brisbane, QLD, Australia

**Keywords:** intimate partner violence, adverse birth outcomes, propensity score matching analysis, quasi-experimental study, sub-Saharan Africa

## Abstract

**Background:**

Intimate partner violence (IPV) is a significant public health problem, with serious consequences on women's physical, mental, sexual, and reproductive health, as well as birth outcomes. Women who encounter IPV are more likely to experience adverse birth outcomes such as low birth weight, premature delivery, and stillbirth. Although numerous studies are exploring the association between IPV and adverse birth outcomes, they merely used classical models and could not control for potential confounders. The purpose of this study was to ascertain whether there was a causation between IPV and adverse birth outcomes in sub-Saharan Africa (SSA) using a quasi-experimental statistical technique [i.e., propensity score matching (PSM) analysis].

**Method:**

This study used the most recent (2015–22) Demographic and Health Survey (DHS) data from 20 SSA countries. A total weighted sample of 13,727 women was included in this study. IPV (i.e., sexual, physical, emotional, and at least one form of IPV) was the exposure/treatment variable and adverse birth outcomes (preterm delivery, low birth weight, stillbirth, and macrosomia) were the outcome variables of this study. PSM was employed to estimate the impact of IPV on adverse birth outcomes.

**Results:**

The average treatment effects (ATE) of sexual, physical, emotional, and at least one form of IPV were 0.031, 0.046, 0.084, and 0.025, respectively. Sexual, physical, emotional, and at least one form of IPV increased adverse birth outcomes by 3.1%, 4.6%, 8.4%, and 2.5%, respectively. Findings from the average treatment effect on treated (ATT) showed that women who experienced sexual, physical, emotional, and at least one form of IPV had an increased risk of adverse birth outcomes by 3.6%, 3.7%, 3.3%, and 3.0%, respectively, among treated groups.

**Conclusion:**

This study demonstrates a causal relationship between IPV and adverse birth outcomes in SSA countries, indicating a need for programs and effective interventions to mitigate the impact of IPV during pregnancy to reduce related adverse pregnancy outcomes. Furthermore, we suggest further research that investigates the causal effect of IPV on adverse birth outcomes by incorporating additional proximal variables not observed in this study.

## 1 Introduction

Intimate partner violence (IPV) is a behavior that results in physical, sexual, or psychological harm in an intimate relationship and it is a significant public health problem, with a serious impact on women's physical, mental, sexual, and reproductive health (1, 2). Globally, 27% of reproductive-age women have been subjected to some form of physical, and sexual violence by their intimate partners (3). In sub-Saharan Africa (SSA), 12.60%, 30.58%, 30.22%, and 42.62% of women experience sexual, physical, emotional, and at least one type of IPV, respectively (4).

Regardless of whether IPV happens during pregnancy, it can increase the likelihood of experiencing physical and mental health problems for both the woman and her child, and linked to adverse pregnancy outcomes (5, 6). Women who encountered IPV are more likely to experience adverse birth outcomes such as low birth weight, premature delivery, and stillbirth (2, 7–9). However, despite sexual and emotional IPV have serious consequences on the health of mothers (10) and their children, previous studies focused on the link between physical IPV and adverse birth outcomes (11, 12).

Understanding the link between different forms of IPV and adverse pregnancy outcomes may have significant clinical and public health implications, including action to stop violence against women and early detection and treatment.

Moreover, numerous studies on the association between IPV and adverse birth outcomes merely used classical models, which are unable to control for potential confounders. The purpose of this study was to ascertain whether there was a causal relation between different forms of IPV and adverse birth outcomes for women in SSA who recently gave birth to a live child using propensity score matching (PSM) analysis. PSM is a quasi-experimental technique in which each treated unit is matched with a non-treated unit that has similar characteristics and creates an artificial control group. The socioeconomic and demographic profiles of women who have experienced IPV differ initially. Therefore, conducting a standard regression analysis to assess the association between IPV and adverse pregnancy outcomes yields biased estimates. In comparison to the generic multiple logistic regression models, PSM can control potential confounders even when there is a strong association between IPV and confounders. Moreover, randomizing this study was deemed unethical and impractical due to ethical constraints pertaining to the nature of exposure and outcome variables.

## 2 Methods

### 2.1 Data source, study setting, and population

This study used the most recent (2015–22) DHS data from 20 SSA countries, including Ethiopia, Kenya, Uganda, Tanzania, Burundi, Madagascar, Rwanda, and Zimbabwe (East Africa); Angola, and DR Congo (Central Africa); Malawi, and South Africa (Southern Africa); and Burkina Faso, Cote di viorie, The Gambia, Liberia, Mali, Niger, Serra Leone, and Senegal (West Africa). The DHS used a cross-sectional study design. All reproductive-age women who gave birth within 1 year of the recent survey and were interviewed for experiencing IPV were included in this study. This provides an accurate estimate of IPV by considering those women who did not report experiencing IPV during pregnancy/months before the survey as non-exposed. A total weighted sample of 13,727 women was included in this study.

### 2.2 Outcome variables

Adverse birth outcomes–preterm delivery, low birth weight, stillbirth, and macrosomia–were the outcome variables. A newborn weighing less than 2,500 grams was deemed to be a low birth weight (13) and above 4,000 grams was considered macrosomia (14). Preterm births involved deliveries that occurred before 37 weeks of pregnancy (15). Pregnancy losses that take place after 28 weeks of gestation was considered stillbirth (16). A single outcome variable was produced by adding preterm delivery, low birth weight, stillbirth, and macrosomia. If at least one of the previously specified outcomes occurred during labor and recorded at birth, this represented adverse birth outcomes and recoded as “1”.

### 2.3 Treatment/exposure variables

The exposure variables of this study were IPV and its subtypes as such sexual, physical, and emotional IPV. Sexual IPV is a composite variable and generated by aggregating other related variables (i.e., using physical force to have sexual intercourse when she did not want to, physical force to perform any other sexual acts she did not want to, force with threats or in any other ways to perform sexual acts she did not want to in, and any sexual violence). Exposure to sexual IPV was defined if women experienced at least one of these sexual violence acts and coded as “1”, otherwise “0”. Physical IPV was also a composite variable composed of experiences like being kicked/dragged, strangled/burned, and threatened with a knife/gun, and another weapon. If participants were exposed to at least 1 form of physical IPV, they were coded as “1”, otherwise “0”. Emotional violence was generated from variables like exposure to humiliation, threatening with harm, and insult or made to feel bad by husband. If participants were exposed to at least 1 form of emotional IPV, it was coded as “1”, otherwise “0”. Exposure to at least one form of IPV was determined by adding exposure to the above 3 forms of IPV (i.e., sexual, physical, and emotional IPV) experienced by a woman, with “1” indicating exposure to at least one form of IPV.

### 2.4 Confounding variables

The study included covariates that could influence the relationship between IPV and adverse birth outcomes, but not affected by treatment variables (IPV and its subtypes). These included age, marital status, and occupation of the respondent, as well as education and occupation of husband. Moreover, sex of household head, media exposure, place of residence, and household wealth index were considered as confounding variables.

### 2.5 Model building and statistical analysis

Model building and statistical analysis were performed by STATA version 18. PSM is a powerful statistical methodology that estimate the effects of causal treatments. PSM was used in this study because it does not require baseline data, matching is nonparametric (i.e., it does not require a functional form of assumptions for the outcome equation), and matching estimators highlight the issue of common support (17). By applying PSM match each treated unit with a non-treated unit with comparable characteristics (18). To do so, PSM technique applied five basic steps, including propensity score estimation, choosing a matching algorithm, checking common support, checking the quality of matching, and sensitivity analysis (19).

In PSM, propensity score estimation is the initial step. This takes two factors into account–model estimation and variables selection (19). Logit models were applied to predict the probability of participation vs. non-participation. Women who were exposed to IPV and those who did not were matched using logistic regression. The dependent variable in the logit model estimate was IPV and its subtypes, which took a value of “1” if the women were exposed to IPV and its subtypes and “0” if belonged to the non-exposed group. It is mathematically expressed by:

$$p_{i}=\frac{1}{\left(1+e^{\left(B_{0}+B_{1}X_{1}+B_{2}X_{2}+\ldots \ldots .B_{n}X_{n}\right)}\right)}$$

Where *p_i_* indicates the probability of belonging to women exposed to IPV, *e* indicates the base of the natural logarithm, and *X* indicates the explanatory variables. This analysis included variables that affect both IPV and adverse birth outcomes, as only factors including both exposure and outcome variables simultaneously were considered.

Matching algorithms commonly used in PSM involve the Nearest Neighbour (NN), Caliper and Radius, Stratification and Interval, Kernel and Local, and Linear Weighting (19). Unfavorable matches may result from NN matching if the closest neighbor is far away. This can be avoided by setting a tolerance threshold on the largest propensity score distance (caliper). By using NN matching, we were able to match an IPV exposed with a non-exposed person based on propensity scores that were within the 0.015 calipers. The caliper's size was determined by taking 0.2 of the propensity score's logit standard deviation (20).

Only the region of common support (overlap) was used to define average treatment effect (ATE) and average treatment effect on treated (ATT) (19). Therefore, identifying the areas of overlap and common support between the control and treatment groups is crucial. Every observation in this study, that had a propensity score in the opposing group that was greater than the maximum and smaller than the minimum was dropped.

To measure the quality of covariates between participant and non-participant data, pseudo-R^2^ with standardized bias (SB) is used (21). This analysis used the *p*-value of the likelihood ratio and pseudo-R^2^ with SB to evaluate the quality of matching. The Mantel-Haenszel (MH) test statistic was employed to determine if the PSM estimates were vulnerable to hidden bias because the outcome variables were binary (22). Since individuals who appear to be comparable (in terms of confounding) may differ in their odds of receiving the treatment by as much as a factor of 2, we were interested in the sensitivity of the data up to the point where *Γ* = e*^γ^* = 2 (19).

## 3 Results

### 3.1 Characteristics of the participants

A weighted sample of 13,727 women was included in this study, with a median age of 27 [Intre quartile range (IQR): 23–32] years. More than one-fourth, 3,662 (26.67%), women were uneducated, and 865 (23.63%) of them had encountered adverse birth outcomes. The majority [9,703 (70.68%)] of the women were married and 2,152 (22.17%) of them reported adverse birth outcomes. About 5,179 (37.73%) of participants had no media exposure, and one-fourth [1,326 (25.61%)] had adverse birth outcomes. Regarding the wealth index, 2,606 (18.98%) women belonged to the poorest households, of whom 623 (23.89%) had encountered adverse birth outcomes (Table 1).

**Table 1:** Characteristics of participants in 20 SSA (weighted *n* = 13,727).

Variables	Adverse birth outcomes		
	Frequency (%)	Frequency (%)	
		Yes (%)	No (%)
Age (in years)			
15–19	1,130 (8.23)	322 (28.50)	808 (71.50)
20–24	3,516 (25.61)	884 (25.14)	2,632 (74.86)
25–29	3,729 (27.16)	787 (21.12)	2,941 (78.88)
30–34	2,946 (21.60)	642 (21.78)	2,304 (78.22)
35–39	1,729 (12.60)	350 (20.25)	1,379 (79.75)
40–44	589 (4.29)	133 (22.54)	456 (77.46)
45–49	88.65 (0.65)	31 (35.21)	57 (64.79)
Parity			
1–2	5,613 (40.93)	1,367 (24.32)	4,252 (75.68)
3–4	4,568 (33.27)	983 (21.51)	3,585 (78.49)
≥5	3,541 (25.80)	800 (22.59)	2,741 (77.41)
Age at first birth			
<18	5,552 (40.44)	1,327 (23.91)	4,225 (76.09)
≥18	8,176 (59.56)	1,822 (22.29)	6,354 (77.71)
Educational status			
No education	3,662 (26.67)	865 (23.63)	2,796 (76.37)
Primary education	5,009 (36.49)	1,184 (23.63)	3,825 (76.37)
Secondary and higher	5,058 (36.84)	1,101 (21.76)	3,957 (78.24)
Occupation			
Not working	5,120 (34.30)	1,167 (22.79)	3,953 (77.21)
Working	8,608 (62.70)	1,982 (23.03)	6,625 (76.97)
Media exposure			
No	5,179 (37.73)	1,326 (25.61)	3,852 (74.39)
Yes	8,549 (62.27)	6,726 (78.68)	7,823 (21.32)
Wealth index			
Poorest	2,606 (18.98)	623 (23.89)	1,983 (76.11)
Poorer	2,585 (18.83)	657 (25.40)	1,929 (74.60)
Middle	2,756 (20.08)	636 (23.06)	2,120 (76.94)
Richer	2,911 (21.21)	630 (21.64)	2,265 (78.93)
Richest	2,870 (20.91)	605 (21.07)	2,265 (78.93)
Marital status			
Married	9,703 (70.68)	2,152 (22.17)	7,552 (77.83)
Living together	3,428 (24.97)	820 (23.91)	2,608 (76.09)
Widowed	73 (0.53)	20 (27.11)	53 (72.89)
Divorce	158 (1.15)	45 (28.31)	114 (71.69)
Separated	366 (2.66)	114 (31.07)	252 (68.93)
Health insurance			
No	11,705 (91.69)	2,739 (23.36)	8,984 (76.64)
Yes	1,061 (8.31)	199 (18.92)	855 (81.08)

### 3.2 The impact of intimate partner violence on adverse birth outcomes

Unmatched results showed that sexual, physical, emotional, and at least one form of IPV increased adverse birth outcomes in women by 2.8%, 3.0%, 0.6%, and 1.8%, respectively. The average treatment effects (ATE) of sexual, physical, emotional, and at least one form of IPV were 0.031, 0.046, 0.084, and 0.025, respectively. Sexual, physical, emotional, and at least one form of IPV were found to increase adverse birth outcomes by 3.1%, 4.6%, 8.4%, and 2.5%, respectively. The average treatment effect on treated (ATT) result showed that women who experienced sexual physical, emotional, and at least one form of IPV increased the risk of adverse birth outcomes by 3.6%, 3.7%, 3.3%, and 3.0% among treated groups (Table 2).

**Table 2:** A propensity score-matched analysis of the impact of IPV on adverse pregnancy outcomes in SSA.

Treatment variable	Treated	Control	Difference	SE	*T*-test
Sexual IPV					
Unmatched	0.259	0.232	0.028	0.013	2.05
ATT	0.259	0.223	0.036	0.029	−1.21
ATU	0.232	0.262	0.308		
ATE			0.031		
Physical IPV					
Unmatched	0.258	0.228	0.030	0.010	3.10
ATT	0.258	0.221	0.037	0.227	1.62
ATU	0.228	0.277	0.048		
ATE			0.046		
Emotional IPV					
Unmatched	0.239	0.233	0.006	0.009	0.66
ATT	0.239	0.307	0.033	0.331	1.00
ATU	0.233	0.285	0.052		
ATE			0.084		
At least one form of IPV					
Unmatched	0.246	0.228	0.018	0.008	2.25
ATT	0.246	0.216	0.300	0.021	1.44
ATU	0.228	0.251	0.231		
ATE			0.025		

### 3.3 Common support

ATT and ATE are only defined in the region of common support. When using PSM to examine the effects of physical and at least one form of IPV on adverse birth outcomes, 1 observation was eliminated because of common support. For sexual and emotional IPV, no dropped observation since no issues related to common support. This proves the validity of the common support assumption.

### 3.4 Quality of matching

Standard bias and pseudo-R^2^ were used to assess the quality of matching. The mean SB reduction after matching is 23.6%, 16.9%, 13.1%, 9.7%, and 13.5% for sexual, physical, emotional, and at least one form of IPV. This shows a good level of covariate balancing. The pseudo-R^2^ estimates dropped from 0.013 to 0.001 for sexual IPV, 0.248–0.001 for physical IPV, 0.300–0.004 for emotional IPV, and 0.050 to <0.001 for at least one form of IPV after matching (Table 3). Thus, the covariates had low explanatory power after matching for selection into the treatment group. The *p*-values of the likelihood ratio Chi-square suggested no systematic differences in the distribution of covariates between treatment and control cases after matching. The *p*-value after matching was not significant for all of the treatment variables. Thus, the hypothesis that both groups had the same distribution in the covariates after matching could not be rejected.

**Table 3:** Covariate balancing test results for the PSM.

Treatment variable	Pseudo R^2^	Likelihood ratio Chi-square	*p*-value of likelihood ratio	Bias reduction
Sexual IPV				
Unmatched	0.013	95.38	<0.001	23.6%
Matched	0.001	3.52	0.833	
Physical IPV				
Unmatched	0.248	102.06	<0.001	16.9%
Matched	0.001	4.12	0.766	
Emotional IPV				
Unmatched	0.300	42.51	<0.001	9.7%
Matched	0.004	2.56	0.922	
At least one form of IPV				
Unmatched	0.050	84.61	<0.001	13.1%
Matched	<0.001	3.98	0.782	

### 3.5 Sensitivity analysis

To examine if the PSM estimates were sensitive to the hidden bias, the Mantel-Haenszel test statistic was employed. The Q_MH_ test statistic yielded the same result when no hidden bias (*Γ* = 1) was assumed, suggesting a strong treatment effect (Table 4). In a study free of hidden bias, i.e., where *Γ* = 1, the Q_MH_ test statistic is 1.20, 1.97, 2.57, and 0.81 for sexual, physical, emotional, and at least one form of IPV, indicating the matching was free of hidden bias and would constitute strong evidence that IPV caused adverse birth outcomes.

**Table 4:** Sensitivity analysis for the impact of at least one form of IPV on adverse birth outcomes in 20 SSA countries.

Gamma (*Γ*)	Test statistics		Significance level	
	Overestimation (Q_mh+)	Underestimation (Q_mh−)	Overestimation (p_mh+)	Underestimation (p_mh−)
1	0.81	0.81	0.21	0.21
1.05	0.20	1.42	0.42	0.08
1.1	0.30	2.00	0.38	0.02
1.15	0.86	2.56	0.20	0.01
1.2	1.39	3.09	0.08	<0.001
1.25	1.90	3.61	0.03	<0.001
1.3	2.39	4.10	0.008	<0.001
1.35	2.87	4.58	0.002	<0.001
1.4	3.33	5.03	<0.001	<0.001
1.45	3.77	5.48	<0.001	<0.001
1.5	4.19	5.91	<0.001	<0.001
1.55	4.6	6.32	<0.001	<0.001
1.6	5.01	6.73	<0.001	<0.001
1.65	5.39	7.12	<0.001	<0.001
1.7	5.77	7.50	<0.001	<0.001
1.75	6.14	7.87	<0.001	<0.001
1.8	6.50	8.23	<0.001	<0.001
1.85	6.85	8.58	<0.001	<0.001
1.9	7.19	8.92	<0.001	<0.001
1.95	0.81	0.81	<0.001	<0.001
2	0.20	1.42	<0.001	<0.001

Q_mh+, Mantel-Haenszel test statistic given that we have overestimated the treatment effect; Q_mh-, Mantel-Haenszel test statistic given that we have underestimated the treatment effect; p_mh+, level of significance for Q_mh+; p_mh+, level of significance for Q_mh-.

## 4 Discussion

This study used a quasi-experimental technique, namely the PSM to examine the impact of IPV on adverse birth outcomes and to control for the effect of confounding variables. The analysis also used caliper and radius matching with a maximum propensity score distance of 0.15 to compare adverse birth outcomes in individuals who were exposed to IPV with non-exposed. By establishing a suitable comparison group, this strategy is useful for evaluating the effect of a particular intervention in observational studies where randomization is not feasible.

Exposure to sexual, physical, emotional, and at least one form of IPV were found to lead to adverse birth outcomes in the 20 SSA countries. The ATE of those exposed to sexual, physical, emotional, and at least one form of IPV indicated that there was an increased risk for adverse birth outcomes for women who were exposed to the different forms of IPV. Similarly, the ATT of women who experienced these forms of IPV increased the risk of adverse birth outcomes among treated groups. This implied that women who had not sexually, physically, emotionally, or any form of violence by their intimate partner had lower adverse birth outcomes.

Women exposed to sexual IPV had increased adverse birth outcomes. This result is supported by studies from Iran (23), Bangladeshi (24), and Norway (10). This might be due to exposure to IPV during pregnancy might increase the risk of injury to the uterus and leads to a premature baby (25). The other possible reason could be exposure to a serious infection due to forceful sexual act (26). Women who were exposed to physical IPV had increased adverse birth outcomes consistent with other studies (23, 24). Women exposed to blunt physical trauma reported inadequate nutrition, poor access to prenatal care, isolation, negative coping mechanisms, and elevated levels of physical or psychological stress (27). Moreover, any forms of IPV during pregnancy is associated with pregnancy-specific behaviors and exposed women are more likely to miss prenatal care appointments or initiate prenatal care later than recommended (28).

Similarly, women who experienced emotional IPV reported increased adverse birth outcomes, converging with findings from other studies (23). The increased production of cortisol due to related stress may lead to a reduction in uteroplacental blood flow, thus less food and oxygen flow to the fetus, in turn, increasing risk for adverse birth outcomes (29). Exposure to at least one form of IPV also increased the risk of adverse birth outcomes comparable to studies from other low-income countries (2, 30–34).

Overall, these findings have established a causal relationship between IPV and adverse pregnancy outcomes in various SSA countries by estimating the actual effect of sexual, physical, emotional, and at least one form of violence on adverse pregnancy outcomes.

### 4.1 Strengths and limitations of the study

This study used nationally representative data with a large sample size to enhance the generalizability of the findings. Employing PSM enables to balance women exposed to IPV (treatment group) on confounding factors to enhance comparability using a non-experimental causal inference technique. The findings have implications for stakeholders to design appropriate strategies and related services that address IPV and its consequences. Some limitations included potential missing on some data related to adverse birth outcomes since this study used secondary data. Some limitations included potential missing on some data related to adverse birth outcomes since this study used secondary data. There is also a chance of residual confounding because the matching was done solely using the observed factors.

## 5 Conclusion

This study demonstrates a causal relationship between IPV and adverse birth outcomes in SSA countries, indicating a need for programs and effective interventions to mitigate the impact of IPV during pregnancy to reduce related adverse pregnancy outcomes. Furthermore, we suggest further research that investigates the causal effect of IPV on adverse birth outcomes by incorporating additional proximal variables not observed in this study.

## Data Availability

Publicly available datasets were analyzed in this study. This data can be found here: https://data.dhsprogram.com.
